# Chemi- vs physisorption in the radical functionalization of single-walled carbon nanotubes under microwaves

**DOI:** 10.3762/bjnano.5.63

**Published:** 2014-04-29

**Authors:** Victor Mamane, Guillaume Mercier, Junidah Abdul Shukor, Jérôme Gleize, Aziz Azizan, Yves Fort, Brigitte Vigolo

**Affiliations:** 1 Laboratoire SRSMC UMR CNRS 7565, Université de Lorraine, 54506 Vandoeuvre-les-Nancy, France; 2 Institut Jean Lamour, CNRS-Université de Lorraine, BP 70239, 54506 Vandœuvre-lès-Nancy, France; 3 School of Materials and Mineral Resources Engineering, Universiti Sains Malaysia, 14300 Nibong Tebal, Penang, Malaysia; 4 Laboratoire de Chimie Physique-Approche Multi-échelle de Milieux Complexes-Université de Lorraine, 1 Bd Arago, 57078 Metz, France

**Keywords:** carbon nanotubes, covalent functionalization, grafting, microwaves, physisorption

## Abstract

The effect of microwaves on the functionalization of single-walled carbon nanotubes (SWNTs) by the diazonium method was studied. The usage of a new approach led to the identification of the strength of the interaction (physical or chemical) between the functional groups and the carbon nanotube surface. Moreover, the nature (chemical formula) of the adsorbed/grafted functional groups was determined. According to thermogravimetric analysis coupled with mass spectrometry and Raman spectroscopy, the optimal functionalization level was reached after 5 min of reaction. Prolonged reaction times can lead to undesired reactions such as defunctionalization, solvent addition and polymerization of the grafted functions. The strength (chemi- vs physisorption) of the bonds between the grafted functional groups and the SWNTs is discussed showing the occurrence of physical adsorption as a consequence of defunctionalization after 15 min of reaction under microwaves. Several chemical mechanisms of grafting could be identified, and it was possible to distinguish conditions leading to the desired chemical grafting from those leading to undesired reactions such as physisorption and polymerization.

## Introduction

Carbon nanotubes (CNTs) are recognized to have a huge potential in a variety of applications such as electronics, composite materials, energy storage and medicine [1–4]. From bulk synthesis method, CNTs are often entangled contingent upon the production process. They have a high tendency to remain aggregated and are difficult to process if no particular treatment is used to maintain them in a dispersed state. Covalent functionalization is the attachment of a chemical group able to disperse, compatibilize or induce a particular activity to the CNTs. It is recognized to be an efficient way to confer specific surface properties [5]. However, the methods generally used for the covalent functionalization of CNTs often require long reaction times (from several hours to days) [6]. The reaction times can be considerably reduced to a few minutes by using microwave-induced heating [7] as shown by the increasing number of publications in the past years [8]. Microwave irradiation has been efficiently used to assist CNT functionalization by cycloaddition [9–14], aryl radical addition [15–18], oxidation [19], bromination [20] and alkylation [21–22]. Control of the CNT functionalization level is of crucial importance in order to achieve the best benefit of CNT properties in materials and devices. High functionalization levels might lead to both a strong damage of the CNT structure and a disruption of the conjugated π system of CNTs thereby having a negative impact on their intrinsic properties (conductivity, mechanical properties) [23–25]. Low functionalization levels are indisputably preferred for CNT based composites [26].

As a consequence of the fast reaction times achieved under microwave heating, a careful control of the functionalization level has to be performed. Indeed, although the debate on the “microwave effect” in organic chemistry is still open, Kappe et al. have demonstrated that the temperature in the microwave vessel was often underestimated [27].

Therefore, high temperatures and prolonged reaction times can induce a detachment of the functional groups from the CNT surface [15,28–29] or allow the removal of metallic and amorphous carbon impurities resulting in the efficient primary purification of CNTs [30].

**Scheme 1:**
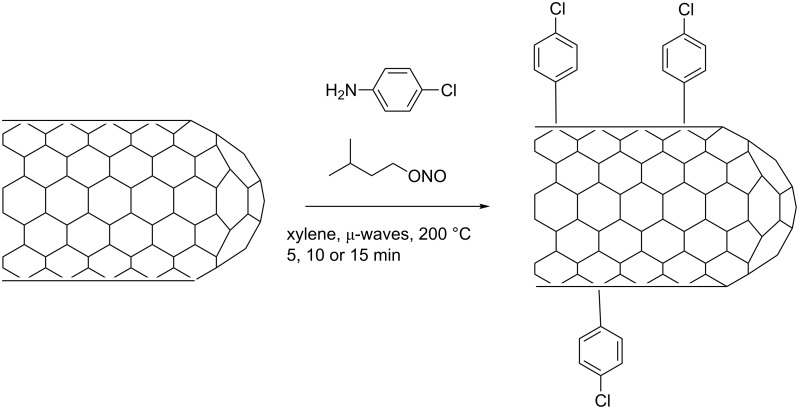
SWNT functionalization by diazonium addition under microwave heating.

With the aim of optimizing a CNT functionalization approach based on microwave-assisted diazonium chemistry [31–32], chlorophenyl groups were grafted at the CNT surface and the functionalization level was followed by thermogravimetric analysis coupled with mass spectrometry (TGA–MS) (Scheme 1).

The present work highlights the importance of controlling the reaction times under microwave heating. Because of the locally high temperatures attained in the microwave reactor, we show that undesired reactions can occur after prolonged reaction times such as the addition of the solvent xylene, partial functional group detachment, and polymerization by adding diazonium to the functional groups already present at the CNT surface.

## Results and Discussion

**Figure 1:**
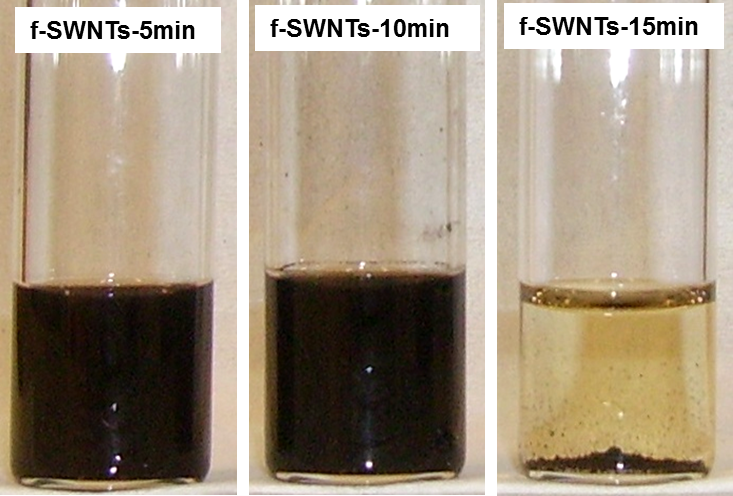
Photographs of f-SWNT-5min, f-SWNT-10min and f-SWNT-15min dispersed in THF 1 week after their preparation.

**Occurrence of the functionalization.** The functionalization was carried out by treating the raw SWNTs with 4-chlorobenzenediazonium (in situ formed by reacting 4-chloroaniline with isoamyl nitrite) under microwaves at 200 °C for 5 min, 10 min and 15 min (Scheme 1). After treatment, the obtained functionalized samples (f-SWNT-5min, f-SWNT-10min and f-SWNT-15min) were analyzed by dispersion tests, high resolution transmission electron microscopy (HRTEM), Raman spectroscopy, and TGA–MS. Figure 1 shows photographs of the dispersions of the f-SWNTs in tetrahydrofuran (THF).

The SWNTs functionalized under 5 min or 10 min of microwaves could be well dispersed leading to dark solutions. As the functionalization duration was increased to 15 min, f-SWNT-15min was very poorly dispersed as a consequence of a rapid aggregation and separation from the solvent. Beyond 10 min of reaction time, the affinity of the f-SWNT surface and the solvent was strongly reduced. THF is a polar aprotic solvent which can lead to the formation of weak bonds with polar surface groups such as chlorophenyl. The observed modification of affinity for f-SWNT-15min means that the SWNTs behaved differently during the reaction than those that were functionalized with shorter times.

**Figure 2:**
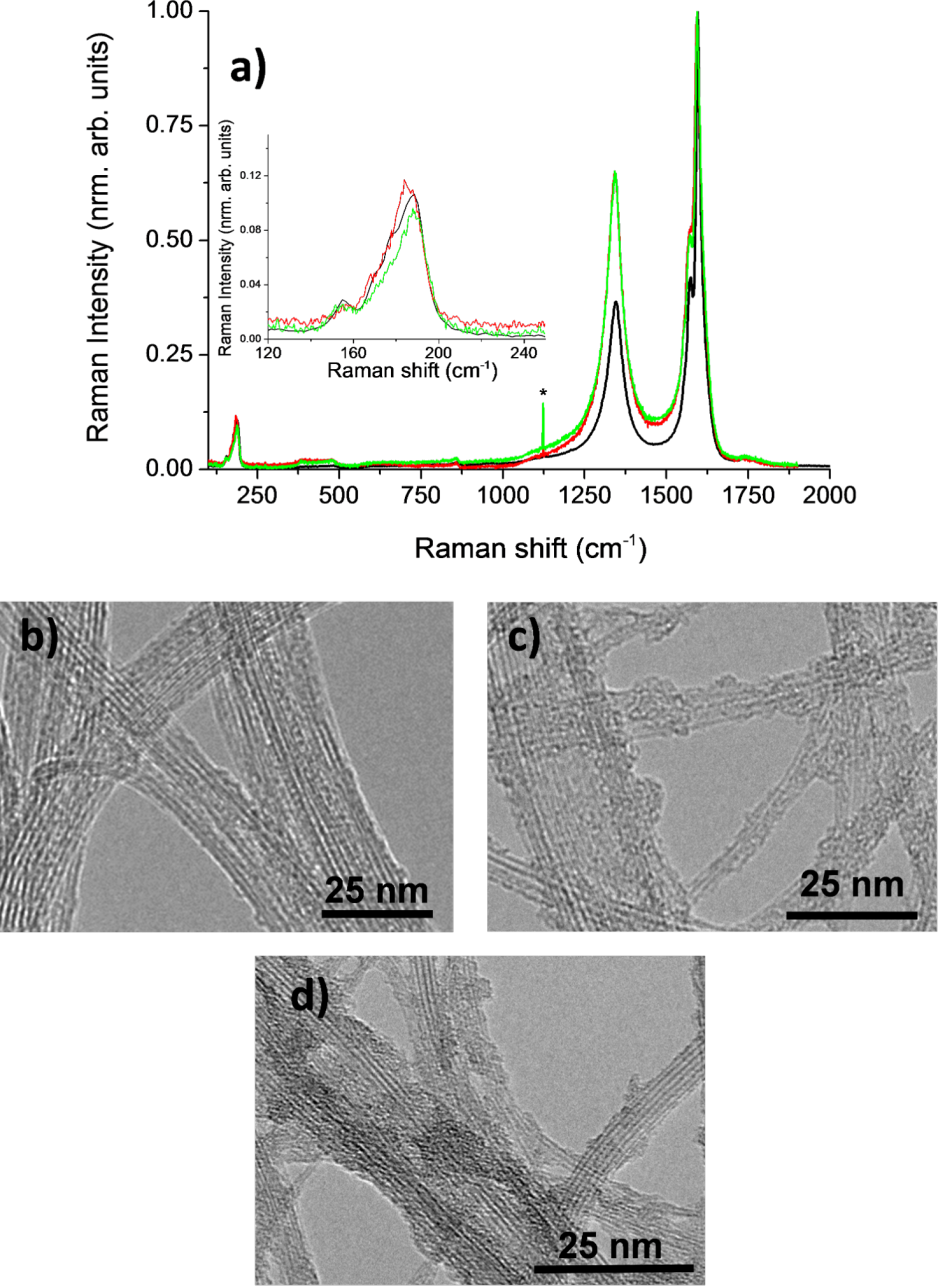
Raman spectra of the starting and functionalized SWNTs and typical HRTEM images. a) Raman spectrum of raw SWNTs (black curve), f-SWNT-5min (red curve) and f-SWNT-10min (green curve).* mercury peak of a reference neon light. HRTEM images of the raw SWNTs (b), f-SWNT-10min (c), and f-SWNT-15min (d).

Raman spectroscopy is a widely used technique to follow the modification of the SWNTs upon a chemical treatment since the D band is sensitive to the introduction of defects in the SWNT sp^2^ structure [31,33]. Figure 2a shows typical Raman spectroscopy spectra of the raw SWNTs (black curve), f-SWNT-5min (red curve) and f-SWNT-10min (green curve).

The recorded signal of f-SWNT-15min shows an intense broad fluorescence band in the domain of interest, so that Raman features could not be observed for this sample. For the two other functionalized samples, the D band intensity is increased after the functionalization reaction due to the induced break of the conjugated structure of the SWNTs in accordance with the expected functionalization mechanism. The *I*_D_/*I*_G_ ratio increases from 0.82 for the raw SWNTs to 1.09 and 1.07 for f-SWNT-5min and f-SWNT-10min, respectively.

The Raman spectra of these two functionalized samples are indeed relatively similar. This could be attributed to none or only a little increase of the functionalization level for f-SWNT-10min compared to that of f-SWNT-5min. The RBM band is only slightly modified after functionalization, and the main contribution located around ω_RBM_ = 185 cm^−1^ is observed for all three samples. The corresponding SWNT diameter *d*_t_ was deduced from

[1]
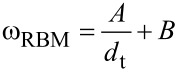


where *A* = 234 nm·cm^−1^ and *B* = 10·cm^−1^ [34]. Following Equation 1 the SWNT diameter *d*_t_ is 1.34 nm as expected for arc-discharge produced SWNTs.

The modification of the SWNT structure caused by functionalization is also revealed in the HRTEM images shown in Figure 2. For the raw sample, the walls of the SWNTs exhibit a low defect level (Figure 2b) and appear undamaged. After functionalization (Figure 2c and 2d), SWNT walls whose damages are difficult to identify in the images are observed. No significant difference between the three functionalized samples (including f-SWNT-5min, not shown in Figure 2) could be evidenced by using HRTEM.

**Figure 3:**
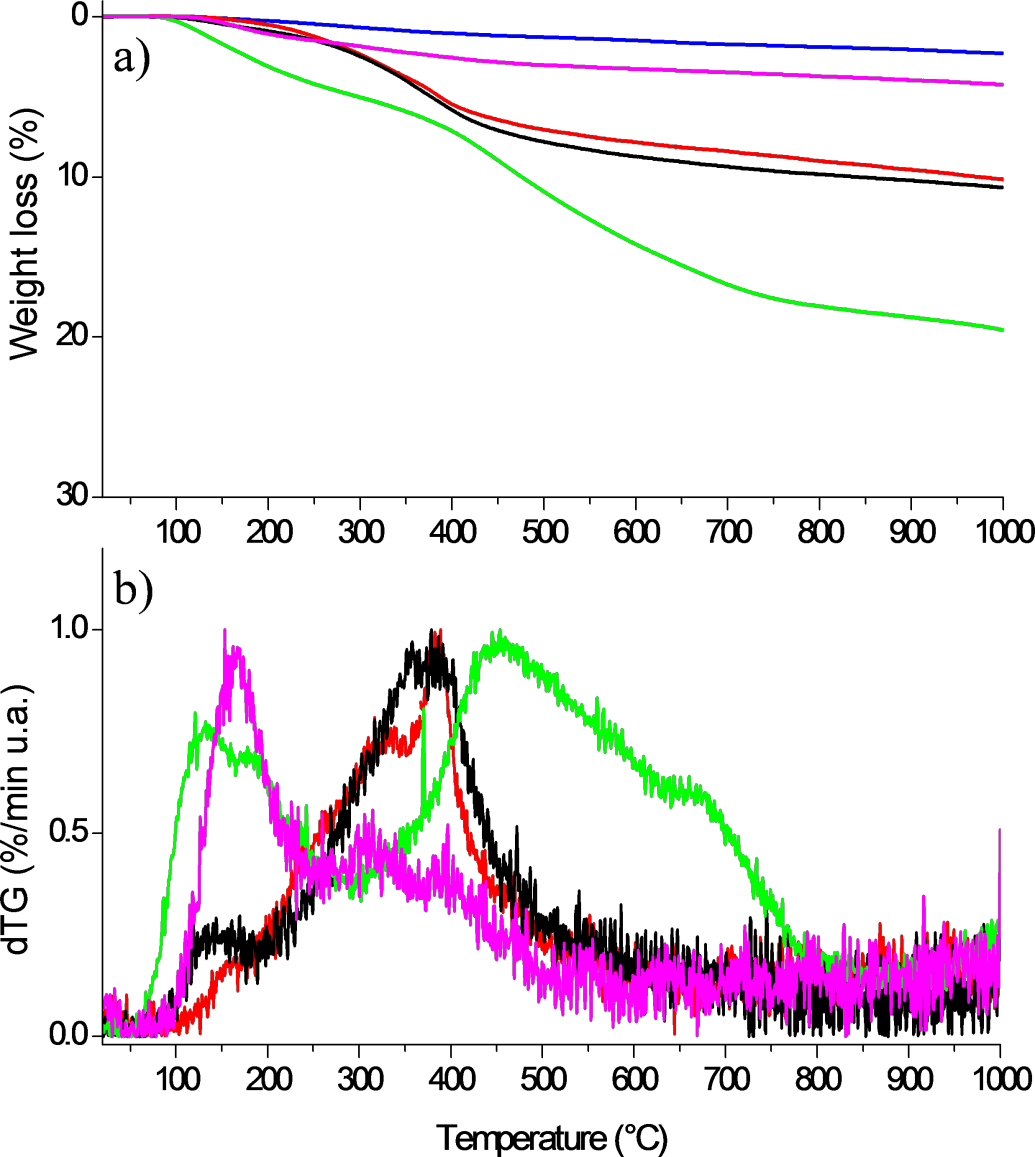
TGA weight loss under helium of raw SWNTs (blue curve), phys-SWNT (violet curve), f-SWNT-5min (red curve), f-SWNT-10min (black curve), and f-SWNT-15min (green curve).

**Functionalization levels and nature of the created bonds.** The recorded weight losses of raw, chemically functionalized samples with three different reaction times and phys-SWNT during heating under helium are shown in Figure 3a. The new sample, phys-SWNT, prepared by mixing the SWNTs with chlorobenzene for 30 min under sonication, was used in order to confirm the covalent nature of the bonds between SWNTs and the chloroaryl group. The derivative representation in Figure 3b helps to position the main weight loss ranges. Consequently, no particular feature is visible in dTG for raw SWNTs (not shown).

**Figure 4:**
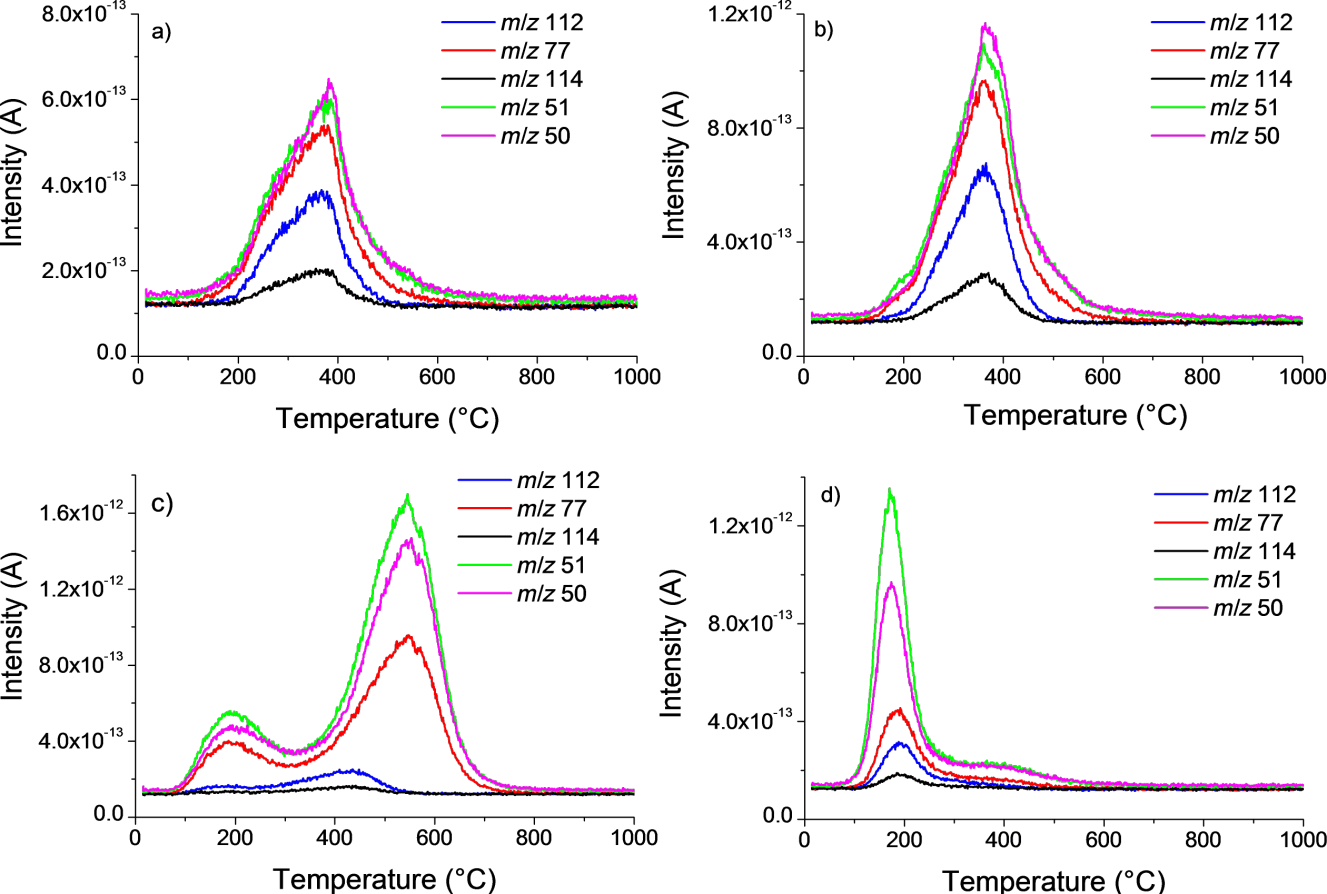
Mass spectrometry intensities for the main *m*/*z* corresponding to chlorobenzene. a) f-SWNT-5min, b) f-SWNT-10min, c) f-SWNT-15min, d) phys-SWNT.

For the phys-SWNT, the weight loss regularly increased above 300 °C identical to the weight loss of the raw SWNTs and is 4.2 wt % at 1000 °C. At a lower temperature, around 174 °C, a more pronounced loss of about 1.5 wt % is attributed to species which are physically adsorbed on the SWNT surface. f-SWNT-5min and f-SWNT-10min show a comparable behavior with a weight loss of 10.1 and 10.7 wt % centered at 380 and 360 °C as the reaction time is increased from 5 to 10 min. The difference between the two samples is a marginal loss (about 1.0 wt %) between 100 and 200 °C only visible for f-SWNT-10min. The TGA profile of f-SWNT-15min is different from the two other functionalized samples. The total weight loss recorded of 19.4 wt % at 1000 °C is almost twice as high. The curve clearly shows two main losses: at a low temperature in range of 100−200 °C and in the range of 400–700 °C with the weight loss being 5.0 and 12.0 wt %, respectively. The sum of the weight losses of these two temperature ranges does not necessarily correspond to the total weight loss recorded at 1000 °C since each of them directly corresponds to the loss recorded in the related temperature range. The lower and upper bounds of the temperature ranges were also determined by means of mass spectrometry data (see Figure 4). As expected, the raw SWNTs bear very few surface groups and thus progressively lost 2.2 wt % during heating under helium.

Table 1 recalls the weight losses corresponding to i) the low temperature range (below 200 °C) assigned to the departure of the groups physisorbed at the sample surface, and ii) the high temperature range (above 200 °C) which typically involves the detachment of covalently functional groups. For the latter, the corresponding functionalization level n considering the exclusive grafting of chlorophenyl groups was determined by using Equation 2:

[2]
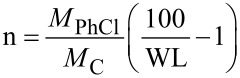


where *M*_C_ and *M*_PhCl_ are the mass of a carbon atom (*M*_C_ = 12 g/mol) and chlorophenyl (*M*_PhCl_ = 111.5 g/mol), respectively. WL (wt %) is the weight loss regarding only the chemisorbed groups. n corresponds to the number of carbon atoms for one functional group.

**Table 1:** Weight loss, temperature ranges for the physisorbed and chemisorbed functional groups, and the corresponding functionalization level.

sample	Total weight loss at 1000 °C /wt %	Chemisorbed functional groups				Physisorbed functional groups	
		Temperature range /°C	Main temperature /°C	Weight loss /wt %	Functionalization level /n	Main temperature /°C	Weight loss /wt %

Raw SWNTs	2.2	—	—	—	—	—	—
f-SWNT-5min	10.1	175–600	380	7.5	114	—	0.0
f-SWNT-10min	10.7	223–586	360	8.0	107	190	1.0
f-SWNT-15min	19.4	325–750	550	12.0	68	190	5.0
Phys-SWNTs	4.2	—	—	—	—	174	1.5

The functionalization level determined for f-SWNT-5min (n = 114) is slightly lower than that obtained for f-SWNT-10min (n = 107) since the weight loss recorded for the latter is higher. The mass spectrometer coupled with the TGA system allows for the examination of the nature of the groups that are detached from the sample surface upon heating. Chlorophenyl groups can be followed by detection of the main fragments expected for pure chlorobenzene, i.e., *m*/*z* 112, 77, 114, 51, 50 with their relative intensity decreasing from 112 to 50. The masses 112 and 114 correspond to the fragments containing chlorine ^35^Cl and ^37^Cl, respectively, whereas the masses 77, 51 and 50 correspond to the fragmentation of the phenyl group from chlorophenyl. Figure 4 shows the detected intensity for the main masses expected for chlorobenzene for f-SWNT-5min (Figure 4a), f-SWNT-10min (Figure 4b), f-SWNT-15min (Figure 4c) and phys-SWNT (Figure 4d).

For the four samples, the *m*/*z* of the expected fragments for chlorobenzene could be detected, and as the intensity profile of each mass is comparable they can be considered without ambiguity as the signature of chlorophenyl groups grafted at the sample surface. Clearly, their departure occurs according to a one-step mechanism for f-SWNT-5min and in two distinct steps for f-SWNT-15min. The behavior of f-SWNT-10min mainly follows a one-step mechanism, but for the most intense masses (77, 51, 50) a low temperature bump begins to appear. For phys-SWNT, the main intensity is located at low temperatures as expected for non-covalently grafted functional groups. Traces of chlorobenzene could also be detected at high temperatures. Still, the intensity of *m*/*z* 112 is less than 1 × 10^−13^ A. For comparison, it is 3.9 × 10^−13^ A for f-SWNT-5min, 6.5 × 10^−13^ A for f-SWNT-10min, and goes down to 2.4 × 10^−13^ A for f-SWNT-15min. For all samples, the relative intensity of the recorded *m*/*z* of chlorobenzene turned out to be different from our expectations with intensities for the fragments containing chlorine (*m*/*z* 112 and 114) being less intense than those from the phenyl part (*m*/*z* 50 and 51) [6]. It is noteworthy that this difference is more pronounced for f-SWNT-15min.

**Figure 5:**
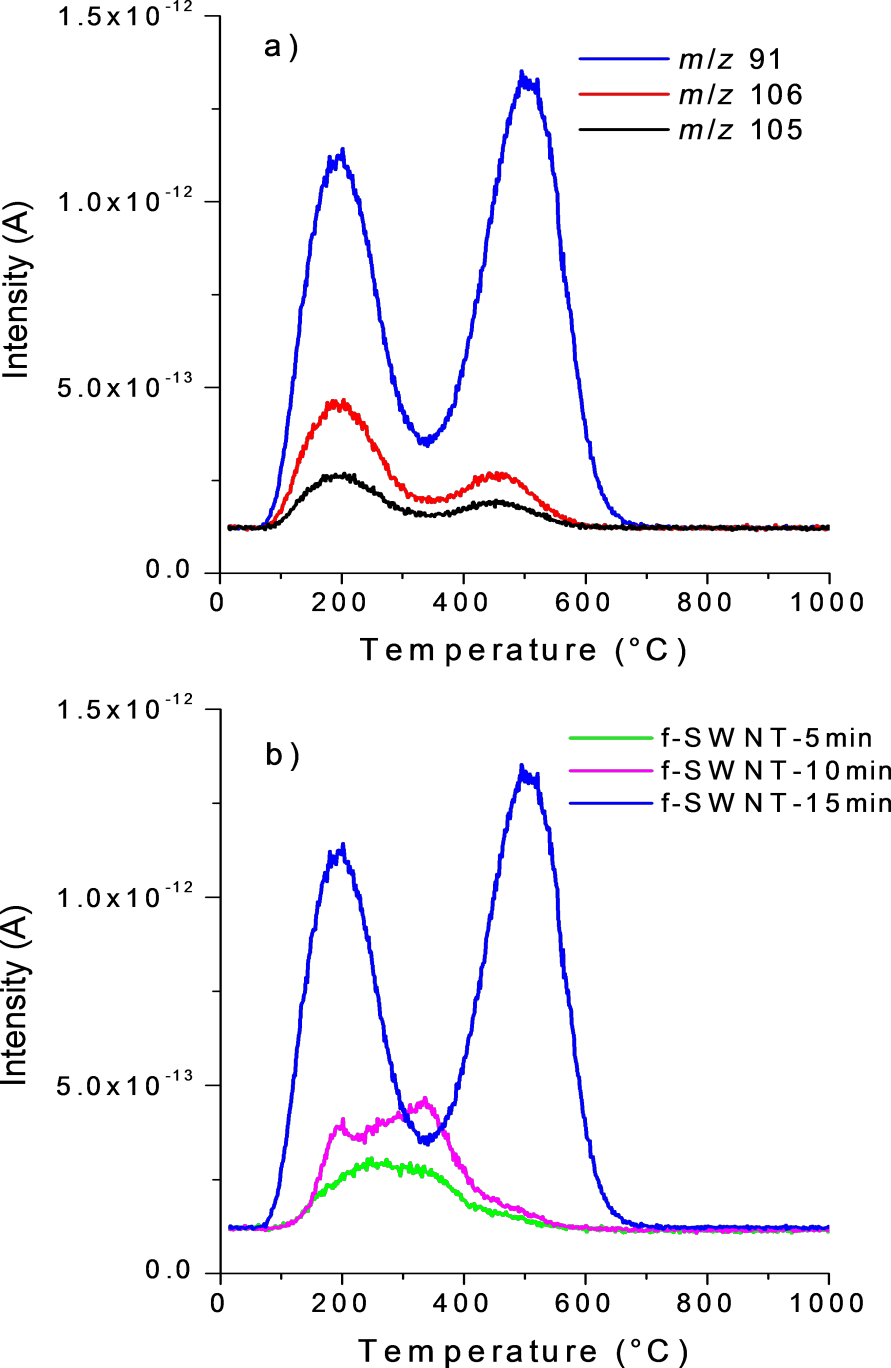
Mass spectrometry intensities for xylene. a) main *m*/*z* of xylene for f-SWNT-15min, b) *m*/*z* 91 for f-SWNT-5min (pink curve), f-SWNT-10min (green curve), and f-SWNT-15min (blue curve).

**Over-reactions under microwaves and chemical mechanisms.** The main fragments for pure xylene (*m*/*z* 91, 106 and 105) which has been used as the solvent for the reaction were clearly detected for f-SWNT-15min (Figure 5a).

The most intense mass for xylene, *m*/*z* 91, is, as expected, superimposed for the three functionalized samples (Figure 5b). Whereas a broad weak peak appears around 300 °C for f-SWNT-5min, as the reaction time is increased, the intensity of *m*/*z* 91 is amplified and significantly more structured. In particular, this holds true for a contribution emerging at high temperature around 550 °C. Raman and TGA–MS data clearly evidence the covalent functionalization of the chlorophenyl groups at the SWNT surface after a reaction time of 5 min. *I*_D_/*I*_G_ is indeed increased after functionalization due to the opening of the C=C bonds by a radical reaction. From Raman and TGA–MS data, it appears that the level of functionalization is almost identical between a reaction time of 5 and 10 min.

The functional groups grafted at the surface of SWNTs are mainly covalently bonded chlorophenyl groups. However, after 10 min, a small amount of physisorbed chlorophenyl and xylyl groups can be detected. The large temperature range for xylene departure suggests a possible solvent addition as previously observed with toluene during the reaction of arylhydrazines with SWNTs under conventional thermal conditions. The longer reaction time under microwave heating can either induce defunctionalization [15] or it can facilitate the debundling of the SWNTs. The latter ultimately results in the removal of physisorbed functional groups which were trapped in the bundles and therefore not visible in the TGA–MS of sample f-SWNT-5min. After 15 min of reaction, these additional processes (defunctionalization/debundling and xylene addition) are more pronounced. Indeed, in TGA–MS of f-SWNT-15min, the peaks around 200 °C related to physisorbed chlorophenyl and xylyl groups are significantly increased. Another feature of the f-SWNT is the apparent increase of the functionalization level, since one function each 68C can be calculated from the TGA curve for f-SWNT-15min. However, a careful analysis of the TGA–MS profiles for the detachment of the chlorophenyl groups reveals that most of the groups are detected between 350 and 700 °C. The same observation can be made for the detachment of xylyl groups. In this case, however, half of the groups are detected in the range of 100–200 °C and the other half between 350 and 700 °C. These large temperature ranges can be explained by the progressive detachment of functional groups from a polymeric structure. Indeed, under the reaction conditions, the aryl radicals can give rise to the growth of aryl chains at the SWNT surface [35].

**Scheme 2:**
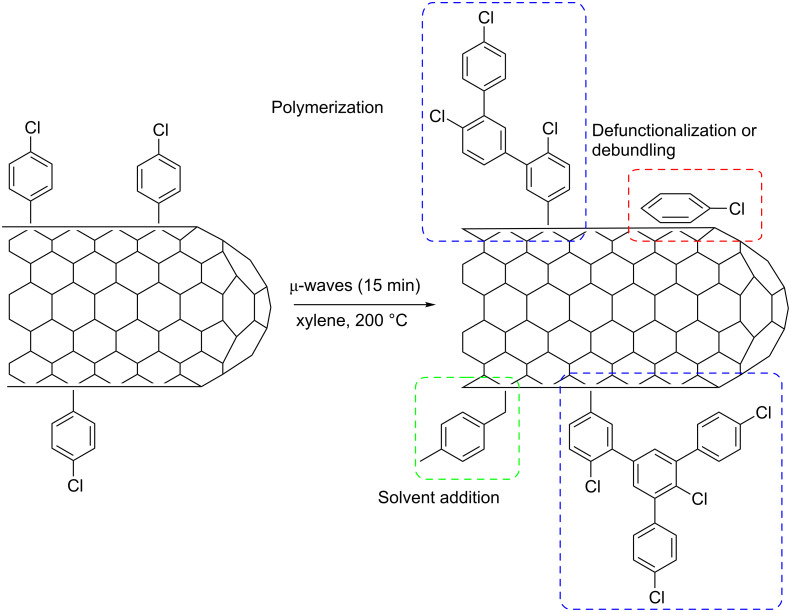
Over-reactions after 15 min under microwaves.

All these possible over-reaction processes are summarized in Scheme 2. The proposed polyaromatic structure for f-SWNT-15min sample can explain the intense broad fluorescence band observed during the Raman experiments as well as the poor dispersion ability in THF compared to f-SWNT-5min and f-SWNT-10min. Moreover, it can explain the much lower detection of *m*/*z* 112 and 114 compared to *m*/*z* 50 and 51 in TGA–MS of sample f-SWNT-15min. Indeed, the analysis of mass spectra of several dichloro-biphenyles revealed that *m*/*z* 50 and 51 are higher compared to *m*/*z* 112 and 114, whereas it is vice versa for chlorobenzene [36].

## Conclusion

SWNTs were efficiently functionalized under microwaves by aryl radical addition. The functionalization level and more importantly the nature of the functional groups could be controlled by performing the reaction in very short time. The chlorophenyl groups covalently grafted at the SWNT surface were detected by TGA-–MS after only 5 min of reaction. The TGA-MS technique also showed that after 15 min of reaction, the functionalization level was increased but the nature of the functional groups as well as the interaction between the functional groups and SWNTs (chemi- vs physisorption) were evolved. Given the reported reaction conditions, longer reaction times under microwaves caused three undesired processes: defunctionalization, solvent addition, and polymerization of the grafted functions.

## Experimental

**Reagents.** Single-walled carbon nanotubes (SWNTs) were obtained by means of a home-made reactor using conventional experimental conditions [37]. SWNTs are used without any purification process because common purification treatments may introduce additional defects on the SWNT surface. 4-Chloroaniline and isoamyl nitrite were purchased from Sigma-Aldrich and used as received. Xylene (mixture of isomers) was purchased from Carlo-Alba and used as received.

**Functionalization procedure.** The microwave device was a CEM discover System. In a 10 mL glass tube, SWNTs (15 mg) were mixed with 4-chloroaniline (300 mg) in xylene (2.5 mL). The mixture was gently sonicated for 10 min until it was visually homogenous. Isoamyl nitrite (0.45 mL) was added and the glass tube was sealed with a Teflon cap. A set of reactions was carried out in the microwave oven at 200 W with different reaction times (5 min, 10 min and 15 min) under cooling conditions to obtain a constant temperature of 200 °C and a pressure of 17 bar during the reaction. After the reaction, the nanotubes were filtered on a FG filter (pore size 0.2 µm) and washed with dimethylformamide (DMF) and methanol several times until the solvent became colorless. Modified SWNTs were dried in vacuum at 80 °C overnight. The obtained samples functionalized with 5, 10 and 15 min of microwaves were named f-SWNT-5min, f-SWNT-10min and f-SWNT-15min.

**Characterizations.** For the dispersion tests, functionalized SWNTs in a powder state were added to tetrahydrofuran (THF) and the mixture was dispersed by using a sonication bath during 15 min. The solutions were allowed to settle for one week. Raman spectra were collected at room temperature (300 K) with a LabRAM HR 800 micro-Raman spectrometer. The incident light from a 514.5 nm laser was focused on the samples with a x50 microscope objective with a power density of 0.25 mW/µm^2^. Three spectra were recorded for the same sample. The main Raman features are the Radial Breathing Modes (RBM) in the 150–300 cm^−1^ range. At higher frequency, the D band, which is located in the 1320–1350 cm^−1^ range, and the G band in the 1500–1600 cm^−1^ range correspond to the C=C bond vibrations of the nanotubes. The D band was fitted with a Lorentzian curve and the G band was fitted with three Lorentzian curves: the G^−^ band around 1570 cm^−1^, the G^+^ band at about 1594 cm^−1^ and the G* band at around 1614 cm^−1^. The G area is obtained from the sum of the areas of the 3 corresponding components. The calculated areas of the D and G band are used to obtain the intensity ratio *I*_D_/*I*_G_. For the sake of clarity in the figure, the intensities of the spectra were normalized with respect to the maximum of the G^+^ band. For high resolution transmission electron microscopy (HRTEM) observations, SWNTs were dispersed in ethanol in a sonication-bath for a few minutes and deposited on a holey carbon copper grid (300 mesh). A Philips CM 200 apparatus was used at an operating voltage of 200 kV. About 10 zones were observed for each sample in order to obtain a statistical view, and we show one typical image for each sample. A SetaramSetsys evolution 1750 Thermal Gravimetric Analyser coupled with a Pfeiffer GSD 301C Vacuum OmniStar mass spectrometer (TGA–MS) was used for the detection of the detached functions from the SWNT surface. About 5 mg of raw or functionalized sample were placed in an alumina crucible in the TGA chamber, and the temperature was increased from room temperature up to 1000 °C under a helium Alphagaz 2 flux of 20 mL/min at a rate of 3 °C/min. Derivative data (dTG) were obtained by deriving the weight loss with respect to time. The parameters we used for the mass spectrometer ensure that most of the species undergo single ionization, that is, *z* = 1 for the detected *m*/*z*. Thus, *m*/*z* and mass will be interchangeably employed in the text.
